# Hydrolytic enzymes and their directly and indirectly effects on gluten and dough properties: An extensive review

**DOI:** 10.1002/fsn3.2344

**Published:** 2021-05-24

**Authors:** Kiana Pourmohammadi, Elahe Abedi

**Affiliations:** ^1^ Department of Food Science and Technology College of Agriculture Fasa University Fasa Iran

**Keywords:** Enzymatic modification, Gluten, Physicochemical properties, Rheological properties

## Abstract

Poor water solubility, emulsifying, and foaming properties of gluten protein have limited its applications. Gluten is structured by covalent (disulfide bonds) and noncovalent bonds (hydrogen bonds, ionic bonds, hydrophobic bonds) which prone to alteration by various treatments. Enzyme modification has the ability to alter certain properties of gluten and compensate the deficiencies in gluten network. By hydrolyzing mechanisms and softening effects, hydrolytic enzymes affect gluten directly and indirectly and improve dough quality. The present review investigates the effects of some hydrolytic enzymes (protease and peptidase, alcalase, xylanase, pentosanase, and cellulase) on the rheological, functional, conformational, and nutritional features of gluten and dough. Overall, protease, peptidase, and alcalase directly affect peptide bonds in gluten. In contrast, arabinoxylan, pentosan, and cellulose are affected, respectively, by xylanase, pentosanase, and cellulase which indirectly affect gluten proteins. The changes in gluten structure by enzyme treatment allow gluten for being used in variety of purposes in the food and nonfood industry.

## Graphical abstract

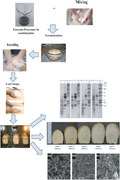

**Abbreviations**
CD: celiac disease
DH: degree of hydrolysis
DSC: differential scanning calorimetry
FDA: food and drug administration
FTIR: Fourier transforms infrared spectroscopy
GRAS: generally recognized as safe
HMW‐GS: high molecular weight‐glutenin subunits
LAB: lactic acid bacteria
LMW proteins: low molecular weight protein
MW: molecular weight
PEP: Prolyl endopeptidases
R_max_
: maximum resistance
SDS‐PAGE: sodium dodecyl sulfate‐poly acryl amide gel electrophoresis
SH: thiol group
SS: disulfide bonds
Tg: glass transition

## 1 INTRODUCTION

Storage proteins existing in wheat gluten structure cause the exclusive viscoelastic characteristics of wheat dough when gluten is hydrated (Delcour et al., 2012). As vital components in wheat endosperm, gluten proteins are well‐known for their use in bread formulation, determining its quality. According to Osborne fractionation procedures, gluten is classified into storage proteins that confer a viscoelastic behavior in bakery products. Composed of gliadins (soluble in 70% ethanol) and glutenins (insoluble in 70% ethanol), gluten proteins are almost insoluble in water.

Glutenin subunits with size range from about 500,000 to more than 10 million (g/mol) are comprised of aggregated proteins formed by SS bonds and are among the largest proteins in nature. The glutenin subunit is categorized into two types, namely LMW‐GS (30,000 to 45,000) and HMW‐GS (70,000 to 90,000) (Abedi & Pourmohammadi, 2020a, 2020b,2020a, 2020b). Gliadins (prolamins), with a monomeric structure, are categorized into four classes, namely α, β, γ, and ω gliadins (Abedi & Pourmohammadi, 2021a). The relative molecular weights of α, β gliadins are about 30,000–40,000 g/mol, while, due to the existence of notable amount of sulfur amino acids, γ gliadins have higher molecular weight than α, β gliadins. In contrast, the ω‐gliadins with size range from 44,000 to 80,000 (g/mol) contain considerable amounts of glutamine/glutamic acid, proline, and phenylalanine and are completely deficient in sulfur amino acids (Majzoobi, et al., 2012). Therefore, the low gluten solubility is due to the small number of ionizable amino acids and high amounts of glutamine, proline, and glycine in gluten construction.

Despite the myriad interesting functionalities of wheat glutens, this protein has poor water solubility, emulsifying, and foaming properties which have limited its applications. There are various methods for improving the functionality of glutenin and gliadin by modifying their structure. As a biotechnological treatment, enzyme modification is one of the methods which extends gluten applications.

**FIGURE 1:**
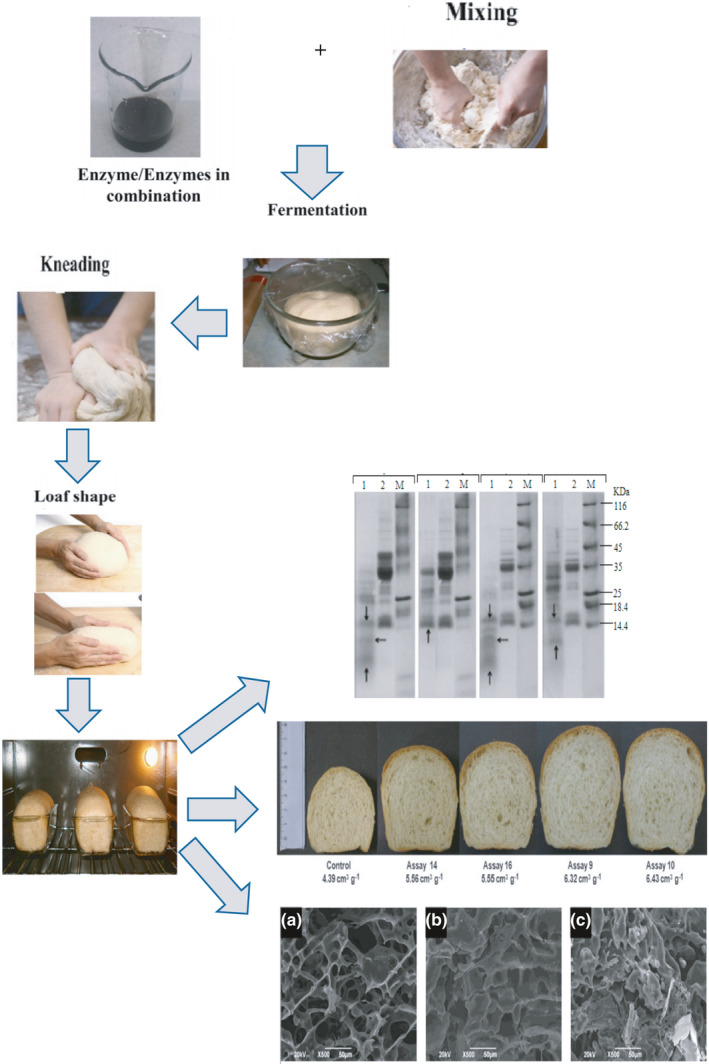
Application of hydrolytic enzymes on dough structure

There are numerous limitations in using flours with strong gluten networks; therefore, the hydrolyzing effects of enzymes allow to use these flours in various purposes. Improvements in bread properties obtained by the addition of hydrolytic enzymes have been associated with their impact on the physical properties of the dough during processing. One of the major functions of hydrolytic enzyme addition is to soften dough to improve machining properties and thus enhance bread quality (Figure 1; Harada et al., 2000; Pourmohammadi & Abedi, 2021; Yong et al., 2006).

The present review investigates the modes of action of some hydrolyzing enzymes and their effects on the functional, rheological, conformational, and nutritional characteristics of gluten and dough.

### 1.1 Protease and peptidase

Wheat gluten hydrolysis to smaller peptides and free amino acids with more hydrophilic polypeptide (Wang et al., 2016; Zhou et al., 2017; Hwang et al., 2016) normally is carried out via peptidase and protease (reaction 1). Hydrolyzing treatment: 1) enhance functional features (solubility, foaming, and emulsifying capacity) (Wang et al., 2016; Wouters et al., 2016; Wouters, Fierens, et al., 2017; Kammoun et al., 2003); 2) improve the safety and nutritional values of gluten protein by reducing the allergenic potential of wheat gluten which cause celiac disease (Elmalimadi et al., 2017; Henggeler et al., 2017; Merz et al., 2015; Merz, Appel, et al., 2016); 3) improve dough handling through managing the viscoelasticity of gluten network and modifying dough rheology; 4) improve the antioxidant activity of hydrolyzed gluten (Abedi & Pourmohammadi, 2020a; Pourmohammadi & Abedi, 2021). The antioxidant properties are the ability to hinder linoleic acid peroxidation inhibition or put out the DPPH (2,2‐diphenyl‐1‐picrylhydrazyl), ABTS (2,2‐azino‐bis (3‐ethylbenzothiazoline‐6‐sulfonic acid)), or other radicals (Elmalimadi et al., 2017; Elmalimadi, 2018; Jin et al., 2016; Wang et al., 2016). In addition, protein hydrolysates are extensively utilized as functional ingredients in food and chemical industries. Some studies, on the other hand, showed that proteolytic enzymes had undesirable impacts on the volume of bread because of the disruption in gluten matrix, particularly glutenin subunits (Kolpakova et al., 2014). Moreover, the combination of hydrolyzing enzymes with different treatments and additives has considerable effects on gluten properties (Table 1, 2).

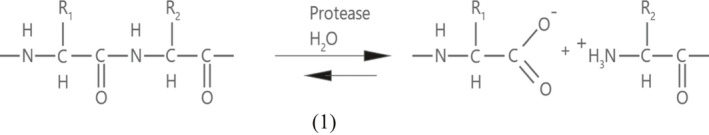

**TABLE 1:** Effects of different treatments in combination with hydrolyzing enzymes on gluten properties

Treatment	Effect	Mechanism	Ref.
Heat treatment +papain	Reduces the free SH in wheat gluten proteins	Makes the structure of wheat gluten more compact	(Wang et al., 2009)
Heat treatment +alcalase	Increases the rate of hydrolysis	Improves gluten susceptibility to alcalase owing to the rearrangements of the inter‐ and intramolecular binding	(Mohamed Bashir Elmalimadi, 2018; Saha et al., 2013)
Heat treatment +alcalase	Enhances the emulsifying properties of gluten	Exposes the hydrophobic protein interior, improving the adsorption at the interface, forming a cohesive interfacial film with the hydrophobic residues	(Mohamed Bashir Elmalimadi, 2018; Phillips & Beuchat, 1981) s
Heat treatment +alcalase	Improves the foaming characteristics	Increases polypeptide chains arising from partial proteolysis, incorporating more air	(Mohamed Bashir Elmalimadi, 2018; Kong et al., 2007a; Wouters et al., 2016)
Heat treatment +alcalase	Increases solubility and water‐holding capacity	Augments cleavable peptide bonds and increases the number of exposed ionizable amino and carboxyl groups	(Mohamed Bashir Elmalimadi, 2018; Hardt et al., 2013)
Heat treatment +alcalase	Reduces the binding affinity to fat	Hydrolytic degradation of the protein structure	(Mohamed Bashir Elmalimadi, 2018)
Heat treatment +alcalase	Increases the DPPH radical scavenging activity (antioxidant ability)	Opens and exposes active amino acid residues, which could react with oxidants or reactive oxygen	(Mohamed Bashir Elmalimadi, 2018; Koo et al., 2014)
Agitation +alcalase	Ameliorates the efficiency of gluten hydrolysis	Reduces the particle size and increases the surface area	(Mohamed Bashir Elmalimadi, 2018)
Temperature (50 ^0^C) + pH 9 + gluten +alcalase	Improves gluten solubility		
Enhances foaming stability of gluten	Reduces the molecular weight and hydrophobicity of wheat protein and increases the content of polar and ionizing groups	(Jakovetić et al., 2015)	
Pancreatin hydrolysis/ Extrusion	Enhances the enzymatic hydrolysis efficiency of wheat gluten	The conformational changes and structural rearrangements of wheat gluten treated with extrusion might modify the catalytic sites of proteases	(Cui, Gong, et al., 2013)

**TABLE 2:** Effects of different additives in combination with hydrolyzing enzymes on gluten properties

Compounds	Effect	Mechanism	Ref.
Starch +flavourzyme + protamex			
	starch granules impede gluten aggregation, which facilitates the hydrolysis	Hinders the gluten aggregation	(Hardt et al., 2015)
Enzymatically hydrolyzed gluten +sucrose	Improves the stability and foaming capacity of hydrolyzed gluten	Increase the affinity of hydrolyzed gluten and adsorption at the water–air interface	(Wouters, Fierens, et al., 2017)
Ethanol +trypsin or pepsin	Increases the foaming capacity and reduces the foam stability of gluten	Alters the air–water interfacial behavior of gluten	(Wouters, Fierens, et al., 2017)
Cysteine +alcalase	Enhances gluten hydrolysis	Alters gluten viscoelastic behavior (varying from more solid‐like to more fluid‐like) and increases its solubility	(Zhang et al., 2012)

### 1.2 Sources of proteases and peptidases for gluten hydrolyzation

Enzymes able to degrade gluten have been detected in various sources including plants (wheat, rye, barley), fungal (*A. niger* and *A. flavus* var. *oryzae*), bacteria (*Flavobacterium meningosepticum, Sphingomonas capsulate*, *Pseudomonas aeruginosa*, *Myxococcus xanthus, Bacillus* sp., *Bifidobacterium* sp., *Lactobacillus* sp., and *Rothia mucilaginosa*), and insects (*Rhizopertha dominica)* (Table 3).

**TABLE 3:** Different sources of proteases and peptidases for gluten hydrolyzation

Type of enzyme	Object	Approach	Ref.
**Plant peptidases**			
Cysteine endopeptidase	Wheat gluten	Attack *N* and C‐terminal sites	(Savvateeva et al., 2015)
Cysteine peptidase	Wheat prolamin	Hydrolyzing wheat prolamin down to 5%	(Gänzle et al., 2008)
Cysteine peptidase	Rye prolamins	Degradation of 99.5% of rye prolamins	(Gänzle et al., 2008)
Triticain‐α	α‐, γ‐, ω‐gliadins and glutenins	Triticain‐α (EC 3.4.22) as a cysteine endopeptidase hydrolyze gluten peptides	(Savvateeva et al., 2015)
Endoprotease B, isoform 2 (EP‐B2)	Barley gluten	Degrade peptide bonds following glutamine, with proline often positioned at the P2	(Savvateeva et al., 2015)
Caricain (EC 3.4.22.30), cysteine endopeptidases papain (EC 3.4.22.2), glutaminyl‐peptide cyclotransferase (EC 2.3.2.5), chymopapain (EC 3.4.22.6)	Wheat gliadin	Caricain is a gluten‐degrading enzyme of the most activity	(Buddrick et al., 2015)
Ginger protease	Wheat gluten	Production of a new type of wheat gluten hydrolysate	(Taga et al., 2017)
**Fungal peptidases (*Aspergillus flavus var. Oryzae)***			
DPP IV (EC 3.4.14.5)	Wheat gluten	Hydrolyze polypeptides and release *N*‐terminal dipeptide	(Merz et al., 2015)
Flavourzyme	Wheat gluten	The addition of flavourzyme to wheat gluten (25 g/L) lead to 9.5 mg residual gliadin/kg hydrolysate	(Eugster et al., 2015; Merz, Kettner, et al., 2016)
**Fungal peptidases (*Aspergillus niger*)**			
Prolyl endopeptidase	α‐gliadins, γ‐gliadins, HMW‐GS, and LMW‐GS	Degrade CD‐active peptides along with intact α‐gliadins, γ‐gliadins, HMW‐GS, and LMW‐GS	(König et al., 2017; Stepniak et al., 2006)
Aspergillopepsin	Wheat gluten	gluten‐hydrolyzing reactions	(Ehren et al., 2009)
*Fusarium graminearum* proteases	Wheat gluten	These proteases were observed to be essentially serine proteases like trypsin cutting the proteins at the lysine or arginine residues	(Koga et al., 2019)
*Fusarium graminearum* proteases	Wheat gluten	*Fusarium graminearum* proteases reduce glutenin amount and increase gliadin	(Eggert et al., 2011)
*Fusarium. poae*	Wheat gliadin	Gliadin degradation	(Brzozowski et al., 2008)
**Fungal peptidases (*Fusarium graminearum)***			
*Aspergillus usamii* protease	Wheat gluten	Increasing the protein hydrolysates solubility resulting from its secondary structure destruction	(Deng et al., 2016)
*Aspergillus usamii* protease	Wheat gluten	The cleavage of peptides by enzyme and unfolding the globular structure of gluten were able to promote the cross‐linking between peptides—lipid, and contribute to anchoring the peptide molecules at the oil–water interface, improving the emulsifying properties and decreasing the interfacial tension	(Deng et al., 2017)
*Aspergillus usamii* protease	Wheat gluten	Increase the water‐holding capacity of wheat gluten/ increase in β‐sheet ratio	(Saberi et al., 2008)
*Aspergillus usamii* protease	Wheat gluten	Increase the oil holding capacity of wheat gluten/ hydrophobic regions, are more exposed to the aqueous phase	(Saberi et al., 2008)
**Bacterial peptidases**			
Peptidase from *B. subtilis* and *B. licheniformis*	Wheat gluten	Hydrolyzing wheat gluten to a degree of 35%–38%	(Stressler et al., 2015)
Thermolysin (EC 3.4.24.27) from *B. thermoproteolyticus,* subtilisin (EC 3.4.21.62) from *B. licheniformis*	Wheat gliadin	Degradation of wheat gliadin to small residues with low molecular weights (<15,000)	(Socha et al., 2019)
Prolyl endopeptidase from *Lb. brevis, Lb. alimentarius*, *Lb. hilgardii*, *Lb. sanfranciscensis*	Wheat gliadin	Hydrolyzing gliadin fractions/the CD patients tolerated the resultant bread	(Saberi et al., 2008)
Dipeptidases (EC 3.4.13), including PepD and dipeptidyl‐ and tripeptidylpeptidases (EC 3.4.14) such as proline‐specific Xaa‐Pro dipeptidyl peptidase (PepX), metalloendopeptidases (EC 3.4.24) like PepO, PepF, and aminopeptidases (EC 3.4.11) such as PepN and PepC	Wheat gluten	Gluten degradation	(Taga et al., 2017)
Prolyl endopeptidase and peptidases from *L. sanfranciscensis* DSM20663, *L. acidophilus* 5e2	ω‐gliadins and HMW‐glutenins	Degradation of ω‐gliadins and HMW‐glutenins	(Gänzle et al., 2008; Nionelli & Rizzello, 2016)
**Insect peptidases**			
Serine‐type carboxypeptidase (EC 3.4.16)/ serine endopeptidases (EC 3.4.21) from *Oryzaephilus surinamensis, Rhizopertha dominica, Tenebrio molitor, Alphitobius diapernius, T. confusum*, and *T. castaneum*	Wheat gluten	Gluten degradation with postproline cleaving patterns	(Mika et al., 2015)
Endopeptidases from *Pentadomidae* and *Lygaidae, Nysius huttoni*	HMW‐GS	The cleavage of peptides by enzyme, unfolding the globular structure of gluten, promoting the cross‐linking between peptides—lipid, contributing to anchoring the peptide molecules at the oil–water interface, lead to improve emulsifying properties and the interfacial tension decrease	(Koksel, 2001)
Endopeptidases from *Eurygaster Aelia, E. Maura, E. integriceps*	Gliadin	Increase the water‐holding capacity of wheat gluten/ increase in β‐sheet ratio	(Sivri et al., 1998)
Endopeptidases from Eurygaster spp, Aelia spp, *E. integriceps*	Glutenin	Increase the oil holding capacity/ enzymatic hydrolysis cause hydrophobic regions, to be more exposed to the aqueous phase	(Yakovenko et al., 1973)
Bug proteolytic enzymes	Glutenin	Reduction in the certain bonds intensity and creation of two new bands in the electrophoretic patterns according to SDS‐PAGE	(Sivri et al., 1998; Yakovenko et al., 1973)

#### 1.2.1 Microbial peptidases

**FIGURE 2:**
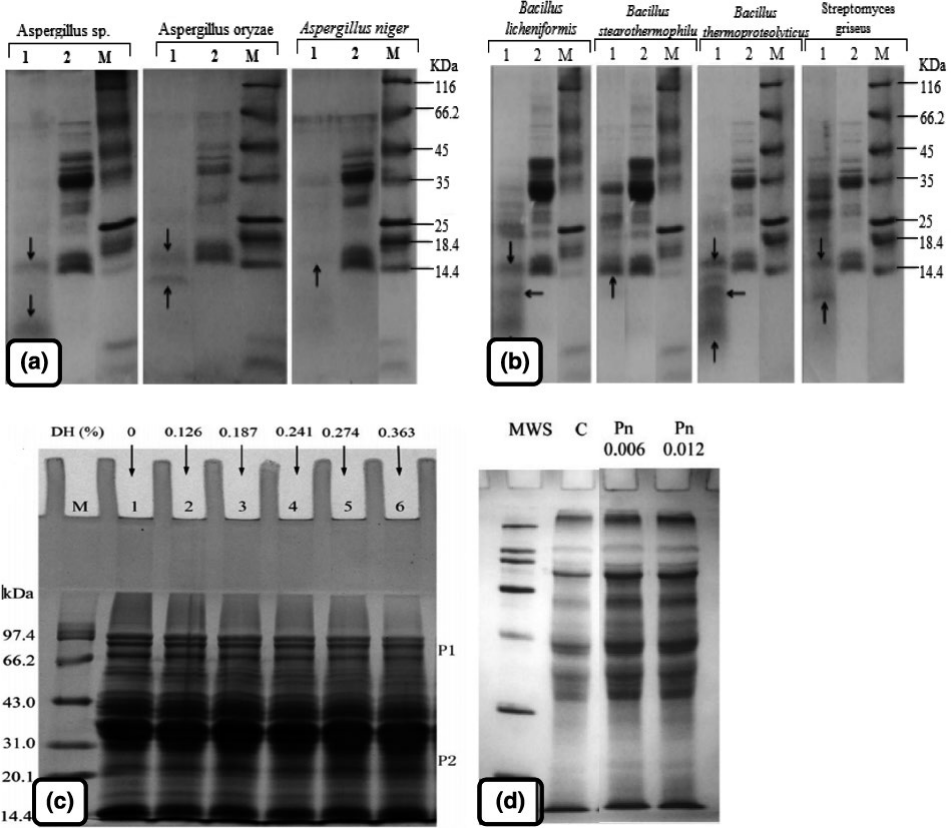
SDS‐PAGE after 60 min of proteolysis of gliadin using specific fungal proteases isolated from *Aspergillus oryzae* and *Aspergillus niger*. Lane 1: wheat gliadins treated using fungal proteases; lane 2: untreated wheat gliadins; M: molecular marker; vertical arrows indicate peptides after hydrolysis (A); SDS‐PAGE for gliadin treated with bacterial proteases isolated from *Bacillus licheniformis*, *Bacillus stearothermophilus*, *Bacillus thermoproteolyticus,* and *Streptomyces griseus*. Lane 1: wheat gliadins treated using bacterial proteases; lane 2: untreated wheat gliadins; M: molecular marker; vertical arrows indicate peptides after hydrolysis (B) (Socha et al., 2020); SDS–PAGE patterns of wheat gluten treated with alcalase. *M* = protein molecular weight marker; 1 = raw wheat gluten; 2–6 = 0.02%, 0.04%, 0.06%, 0.08%, and 0.10% Alcalase‐treated wheat gluten, respectively (C). Electrophoretic patterns of soluble dough treated with 0.006% and 0.012% of pentosanase (D) (Steffolani et al., 2010)

Microbial prolyl endopeptidases (PEPs) are endoproteolytic enzymes which are capable of degrading gluten proteins according to SDS‐PAGE analysis (Figure 2 A and B; Knorr et al., 2016). This could be obtained via fungal (*A. niger*, *A. oryzae,* *A. usamii, F. graminearum*) or bacterial enzymes (*Flavobacterium meningosepticum* (FM), *Sphingomonas capsulata* (SC), *Pseudomonas aeruginosa* (PA), *Myxococcus xanthus* (MX), *Bacillus* sp., *Bifidobacterium* sp., *Lactobacillus* sp., and *Rothia mucilaginosa* (RM)). Peptidases (EC 3.4) generally fall under the hydrolases (EC 3) category, which hydrolyze peptide bonds.

##### Fungal peptidases

*A. niger*, *A. oryzae,* *A. usamii, and F. graminearum* are extensively employed in food and feed processing, regarded GRAS by the U.S. FDA. Belonging to *A. flavus* var. *oryzae*, food‐grade dipeptidyl peptidase IV (DPPIV) (EC 3.4.14.5) is an exopeptidase releasing an N‐terminal dipeptide from polypeptides. Nevertheless, DPP IV alone is not able to effectively degrade gluten. Capable of hydrolyzing various food proteins, flavourzyme is an industrial prepared from *A. flavus* var. *oryzae*. This peptidase is composed of two leucyl aminopeptidases (EC 3.4.11), namely DPP IV and V (EC 3.4.14), three endopeptidases, including neutral peptidase I (EC 3.4.24), neutral peptidase II (EC 3.4.24.39), and alkaline peptidase I (EC 3.4.21.63), and α‐amylase (EC 3.2.1.1) (Merz et al., 2015). Wheat gluten (25 g/L) treated with flavourzyme led to 9.5 mg/kg residual gliadin concentration in the dried hydrolysate, which, through filtration, could be further decreased to around 2 mg/kg (Eugster et al., 2015; Merz, Kettner, et al., 2016). Increase in the degree of hydrolysis (DH) was reported to augment the solubility of hydrolysates obtained from *A. oryzae* with a fungal protease; however, foaming features are seemingly impaired beyond a certain DH (14%) (Drago & González, 2000). Another study reported that to obtain desirable emulsifying and foaming properties, the DH should be even lower, 5% to be exact (Brzozowski, 2016; Kong et al., 2007b).

Prolyl endopeptidase from *A. niger*, which called *Aspergillus niger* prolyl endopeptidase (AN‐PEP) (EC 3.4.21.26), has postproline cleaving activity. It is resistant to digestion by pepsin and active in a pH range of 2–8 with optimum activity at pH 4–5; moreover, AN‐PEP is highly able to effectively degrade CD‐active peptides along with intact α‐gliadins, γ‐gliadins, HMW‐GS, and LMW‐GS (König et al., 2017; Stepniak et al., 2006; Kara et al., 2005; Ahmed et al., 2015). It is also expected that protease therapy with AN enhances the symptoms of nonceliac gluten sensitivity because indigestible gluten‐related proteins have been observed to trigger nonceliac gluten sensitivity (Ido et al., 2018). The enzyme is specifically appropriate for food applications owing to its food‐grade status. AN‐PEP was employed to degrade gluten to levels below 20 mg/kg in wheat starch (Walter et al., 2014), wheat bran (Walter et al., 2014), rye flour and sourdough (Walter et al., 2015), and beer (Knorr et al., 2016). Aspergillopepsin (EC 3.4.23.18) from *A. niger* further showed gluten‐degrading activity; compared with AN‐PEP, however, it was not nearly as substrate‐specific and efficient, hence the fact that it might only be utilized complementary to Endoprotease B, isoform 2 (EP‐B2), or AN‐PEP, for instance (Ehren et al., 2009).

*F. graminearum* proteases are not only able to degrade gluten proteins in the grain itself, but also capable of weakening gluten during dough preparation and resting (Koga et al., 2019). These proteases were observed to be essentially trypsin‐like serine proteases cutting the proteins at the lysine or arginine amino acid (Pekkarinen et al., 2007). The primary destruction of HMW‐GS compared to LMW‐GS is possibly explained by their comparatively higher amount of lysine or arginine. Other studies showed reduced glutenin amount in comparison with increased gliadin following *F. graminearum* infection in wheat grains (Eggert et al., 2011).

*A. usamii* protease affected gluten through increasing the solubility of protein hydrolysates resulting from its secondary structure destruction and the enzymatic release of smaller polypeptide units from the protein (Deng et al., 2016). The cleavage of peptides by enzyme and unfolding of wheat gluten's globular structure were able to promote the interaction between peptides and lipid and contribute to anchoring the peptide molecules at the oil–water interface. This increased the emulsifying activity and decreased the interfacial tension (Deng et al., 2017). The ratio of turns decreased, and the globular structure of gluten was unfolded due to the enzymatic cleavage of peptide chains; therefore, longer β‐sheet chains were generated and the β‐sheet ratio increased (Barth & Zscherp, 2002). *A. usamii* protease slightly increased the water‐holding capacity of wheat gluten from 1.47 to 1.75 g/g; however, after hydrolysis, the holding capacity of oil was significantly increased from 0.92 to 2.91 g/g. This is possibly associated with the enzymatic hydrolysis exposing more hydrophobic regions (originally buried within the wheat gluten) to the aqueous phase (Saberi et al., 2008).

##### Bacterial peptidases

Lactic acid bacteria have a highly convoluted peptidase system (Kunji et al., 1996; M’hir et al., 2012); however, it is not a unique strain possibly possessing the whole pattern of peptidases required to hydrolyze all the potential peptides in which the protein is involved. *B. subtilis* and *B. licheniformis* hydrolyzed wheat gluten to a degree of 35%–38%, revealing extracellular peptidase activities comparable to the commercially available endopeptidase preparation alcalase (Stressler et al., 2015). As a nonspecific bacterial protease, alcalase is primarily achieved from *Bacillus subtilis*. Alcalases are classified to serine protease group that start a nucleophilic assail on the peptide bond via a serine residue at the active site (Apar & Özbek, 2010). Hydrolyzed protein obtained from wheat gluten treated with alcalase possess the maximum degree of hydrolysis (15.8%) values and is more effective in gluten hydrolysis compared with pepsin, pancreatin, neutrase, and protamex (Kong et al., 2007a). Furthermore, thermolysin (EC 3.4.24.27) from *B. thermoproteolyticus* and subtilisin (EC 3.4.21.62) from *B. licheniformis* were also able to effectively degrade wheat gliadin to products with molecular weights <15,000 (Socha et al., 2019, 2020). Subtilisin‐modified samples showed the highest extensive change in the immunoreactivity level of gliadin proteins (Leszczyńska et al., 2012). Di Cagno et al., (2002) reported that sourdough lactic acid bacteria positively affected gliadin peptides. The mixed starter containing *Lb. brevis, Lb. alimentarius*, *Lactobacillus hilgardii*, and *Lb. sanfranciscensis* was reported to almost thoroughly hydrolyze gliadin fractions; as shown by intestinal permeability challenge, the CD patients tolerated the resultant bread (Di Cagno et al., 2004). PEP (Prolyl endopeptidases) from *Myxococcus xanthus* and *Sphingomonas capsulate* (Gass et al., 2007), and *Lactobacillus helveticus* (Chen et al., 2003), resulted in similar properties (gluten detoxification).

#### 1.2.2 Plant peptidases

During germination, gluten proteins are degraded to supply the developing embryo with amino acids and nitrogen. Cysteine endopeptidases (endopeptidases function in the middle of polypeptide chains) attack primary cleavage sites in N and C‐terminal domains. This causes the proteins to unfold the central repetitive domain, in turn cleaved at secondary cleavage sites. Cysteine endopeptidases constitute up to 90% of the total degrading activity, followed by metalloendopeptidases (7%), and serine and aspartic endopeptidases. The resultant peptides are further broken down to amino acids by exopeptidases (exopeptidases function close to the polypeptide chains termination) like serine carboxypeptidases and proline‐specific exopeptidases such as DPP II and IV (EC 3.4.14.2), lysosomal Xaa‐Pro carboxypeptidase (EC 3.4.16.2), and Xaa‐Pro aminopeptidase (EC 3.4.11.9) (Simpson, 2001). Peptidases obtained from germinated rye, wheat, and barley grains and bran effectively degraded epitopes into fragments of <9 amino acids (Geßendorfer et al., 2011). Their activities were dependent on cultivar, germination temperature, cereal species, and pH value during application (Kerpes et al., 2016; Schwalb et al., 2012). Wheat grains germinated with high peptidase activity (approximately 70% of the total activity caused by cysteine peptidases) were utilized as raw materials to ferment sourdough with *Lactobacillus brevis* L62. The combination of sourdough fermentation and germination significantly hydrolyzed wheat prolamin down to <5% (Loponen et al., 2009). Similarly, an approach combined sourdough fermentation and germinated rye, showing more than 99.5% of rye prolamins were degraded to contents of 280 – 430 mg/kg (dry matter) (Gänzle et al., 2008; Loponen et al., 2009). Cereal peptidases have the following upsides: (a) if applied properly, they are stable and highly active, (b) their cleavage specificity is naturally optimized to hydrolyze gluten proteins, (c) they are food‐grade, (d) they are obtainable through such established procedures as malting, (e) they can be integrated into production processes in a relatively facile manner, and (f) they are well accepted by consumers. As far as drawbacks are concerned, the gluten‐degrading activity of cereal extracts was much lower than purified enzymes (Walter et al., 2014) and their activity was inhibited by ethanol (≥ 2%) (Knorr et al., 2016). Taken together, the upsides clearly outweigh the downsides; applying gluten‐degrading cereal peptidases is a promising method for generating high‐quality gluten‐free products derived from gluten‐containing cereals, including gluten‐free and barley‐based beer (Knorr et al., 2016).

#### 1.2.3 Insect peptidases

Because of feeding on cereals, insects, particularly grain pests, probably have endogenous gluten‐degrading enzymes. Among the seven screened beetles, the highest belonged to the proteolytic activity of an aqueous extract from *Oryzaephilus surinamensis, Rhizopertha dominica, Tenebrio molitor, Alphitobius diapernius, T. confusum*, and *T. castaneum* against wheat gluten, showing postproline cleaving patterns. Also identified were one serine‐type carboxypeptidase (EC 3.4.16) and two serine endopeptidases (EC 3.4.21), possibly appropriate for extensively degrading gluten (Mika et al., 2015). Wheat gluten was further reported to be damaged by *Pentadomidae* and *Lygaidae* (Sivri et al., 1998), *Nysius huttoni* (Every et al., 1998) (impacting HMW‐GS), *Eurygaster* and *Aelia* (Paulian, 1980), *E. Maura* (Sivri et al., 1998), *E. integriceps* (Koz’mina & Tvorogova, 1973) (affecting total gluten and gliadin), Eurygaster spp. and Aelia spp. (Every et al., 1998; Sivri et al., 1998), and *E. integriceps* (Yakovenko et al., 1973) (influencing glutenin). To solubilize the nutrients, these insects attack developing wheat kernels, injecting their salivary secretions into the grain. These secretions have strong proteolytic enzymes persisting in the flour after milling and in the kernel following harvest. During the dough stage of the bread making process, the proteolytic enzymes break down the gluten structure. The doughs prepared from bug‐damaged wheat flour are sticky, generating loaves of poor volume and crumb texture (Every et al., 1998). Koz’mina and Tvorogova (1973) observed a reduction (caused by proteolytic action) in the relative intensities of certain bands in the electrophoretic patterns of total unreduced gluten and gliadin; also, two new bands appeared at the low mobility region in the gliadin patterns of wheat damaged by *E*.*integriceps*. Researchers also showed that compared with glutenins, the gliadins had more resistance to bug enzymes (Sivri et al., 1998; Yakovenko et al., 1973).

### 1.3 Effect on functional properties

By using hydrolyzing enzymes, the emulsifying capacity of gluten augments which is ascribe to changes in the secondary structure of wheat gluten (Sun et al., 2019) depending on the degree of hydrolysis and protease activity (Deng et al., 2016). Increasing the emulsifying capacity of gluten in hydrolyzed samples can be explained by the unfolding of wheat gluten's globular structure and peptide bond's disruption by enzymatic modifications. Disruption of peptide bonds could facilitate the interaction among peptides and lipids and increase the availability of peptide residues at the oil–water interface leading to reduced interfacial tension and increased emulsifying activity. Nonetheless, moving further in the proteolytic activity, a reduction occurs in the emulsifying capacity of the hydrolysates due to extensive gluten degradation (Wang et al., 2016). The foaming capacity of hydrolyzed gluten was significantly improved by elevating the surface activity and reducing the surface strain at the water–air interface. This is possibly attributed to the large amount of polypeptide chains having broad molecular weight distributions generated from partial proteolysis, hence incorporating more air in wheat gluten (Deng et al., 2016) and enhancing the flexibility induced by the reactions of SS/SH interchange (Wouters et al., 2016). On the other hand, foam stability was reduced in more extensive hydrolysis due to the increased polypeptide chains unable to make foam air cells stable (Wouters, Fierens, et al., 2017; Wouters et al., 2016; Wouters, Rombouts, et al., 2017). Gluten treated with alcalase was further reported to increase solubility, emulsifying capacity, foaming stability, and foaming capacity owing to produce lower molecular size hydrolysates (Elmalimadi et al., 2017; He et al., 2019; Kong et al., 2007b).

### 1.4 Effect on rheological properties

Deng et al., (2016) revealed that enzymatic hydrolysis is capable of breaking some SS bonds to unfold the globular structure of gluten and new SS bonds are generated between both newly generated SH and original free SH groups to stabilize the structure of smaller peptides. According to SDS‐PAGE profile, the molecular weight of all the wheat gluten hydrolysates is drastically decreased (Figure 2) in the hydrolyzed gluten proteins (Wang et al., 2016). Researchers have revealed that the hydrophobicity of hydrolyzed protein is able to augmented or reduced based on enzyme specificity, hydrolysis situations, protein characteristics, and degree of hydrolysis (Wang et al., 2016). At low enzyme concentrations, the hydrophobicity of hydrolyzed gluten, augmented, which could be due to the hydrophobic amino acid exposure through protein unfolding (Wang et al., 2016). In excessive hydrolyzing treatment, hydrophobicity would decrease following two possible reasons: (1) degradation of some exposed hydrophobic regions; (2) burying the hydrophobic amino groups due to hydrolysis reactions (Zhang et al., 2014). As a regard to FTIR results, adding alcalase to wheat gluten reduces α‐helix and β‐turn conformation (Cui, Gong, et al., 2013). The reduction is due to the intermolecular disulfide bonds breakage and the elevation of β‐sheet and random coil caused by the alcalase hydrolyzation of the β‐turns into random coils (Cui, Gong, et al., 2013). Also, Wang et al. (2016) proposed that an acceptable amount of alcalase‐based hydrolysis unfolded the rigid structure of wheat gluten and increased the β‐sheet content.

Based on researches, alcalase‐based partial hydrolysis broke SS bonds, thereby unfolding the protein conformation of wheat gluten and increase the SH content. However, excess hydrolysis exposed many hydrophobic amino acids and formed aggregates belonging to wheat gluten hydrolysate. Besides, free SH groups might have participation in forming such aggregates (Zhao et al., 2013). The antioxidant activity of gluten hydrolyzed by alcalase was also studied, where the antioxidant activity of wheat hydrolysates has positive correlation with the content of hydrophobic amino acids. In other words, by excess hydrolysis, due to the increase in hydrophobic amino acids, the antioxidant activity of gluten would increase (Zhao et al., 2013).

The impact of peptidase hydrolysis on rheological properties is reported in different researches (Ahmed & Ikram, 2015; Koga et al., 2019). Peptidase hydrolysis reduced storage modulus (G´) (Ahmed & Ikram, 2015) and gluten consistency (Koga et al., 2019) due to gluten degradation and digesting effect of protease. In the gluten samples treated with protease, the glutenin polymer size is notably decreased which leads to reduction in maximum resistance to extension (R_max_). Rheological results reveal that glutenin polymer's size reduction by proteolytic hydrolysis in treated flours destroyed gluten structure (Koga et al., 2019).

The interactions among flour components (proteins, starch, fibers, etc) play key roles on the rheological properties of dough. According to researches, proteases diminish the storage (G΄) and complex (G˝) modulus. The weakening effect of proteases on wheat dough relates to the decrease in resistance to extension observed by Indrani et al., (2003). Proteinase activity affects specially to glutenins, which would alter the elasticity of the gluten complex (Caballero et al., 2007). Dough prepared with high levels of protease felt sticky and weak, which probably accounts for its poor performance. This weakness can be attributed to the hydrolysis of gluten proteins which are known to be the major determinant of dough strength (Harada et al., 2000). The hydrolyzing mechanism of protease enzymes results in degrading proteins as enzyme substrate. Hydrolyzed proteins lead to water binding capacity reduction, consequently excess released water, which cause significant reduction in dough viscosity and production of softer dough with better machining properties (Harada et al., 2000). However, it has also been recognized that over enzyme addition can cause overly soft or sticky dough, resulting in machining problems at the sheeter and rounder that lead to a deterioration in bread quality (Harada et al., 2000). Protease and peptidase can be used in bakery industry via their hydrolyzing mechanism (Table 4).

**TABLE 4:** Applications of hydrolytic enzymes in gluten‐based products and their mode of actions

Enzyme	Substrate	Mode of action	Product	Effect	Ref.
Protease	Gluten	Degrading proteins, water binding capacity reduction, excess released water, reduction in dough viscosity, and production of softer dough	Wheat dough	Diminish the storage (G΄) and complex (G*) modulus, weakening effect, decrease in resistance to extension	(Caballero et al., 2007; Harada et al., 2000; Indrani et al., 2003)
Bacterial peptidase/ fungal peptidase/ prolyl endopeptidases (PEPs)	Gliadin	Hydrolyze gliadin into harmless peptides	Wheat gluten	Decrease the gluten concentration and produce safe gluten‐base products for celiac disease	(Heredia‐Sandoval et al., 2016; Scherf et al., 2018; Socha et al., 2019; Wei et al., 2018; G. Wei et al., 2020).
Prolyl endopeptidases (PEPs)	Gliadin	Hydrolyze gliadin into harmless peptides	Beer	Produce safe beer for celiac disease	(Guerdrum & Bamforth, 2012)
Xylanase	Arabinoxylans	Break glycosidic linkages in arabinoxylans, viscosity reduction, Viscosity reduction, polymer chains get next to each other easier, and the gluten aggregation occurs	Bread	Softening effect on dough, release of excess water, viscosity reduction of dough, better machining properties	(Amiri et al., 2016)
Pentosanase	Pentosans	Conversion of water‐unextractable arabinoxylans to water‐extractable forms	Wheat dough	increasing amount of soluble pentosans and released amount of free water and the inhibition of gluten network formation according to glutenin–pentosan interactions, production of weak doughs	(Primo‐Martin et al., 2003)
Cellulase	Cellulose	hydrolyze cellulose into cellobiose, glucose, and oligosaccharides	Bread	Decrease in bread hardness and staling, ameliorate the bread sensory evaluations	(Altınel & Ünal, 2017; Nigam, 2013; Park et al., 2019; Wang, Chen, et al., 2018; Yurdugul et al., 2012
Cellulase	Cellulose	Hydrolyze cellulose into cellobiose, glucose, and oligosaccharides	Cracker	Softening effect on dough, shorter baking time	(Carson, 2017)
Cellulase	Cellulose	Hydrolyze cellulose into cellobiose, glucose, and oligosaccharides	Steamed bread	Elevate bread sensory evaluation	(Lu et al., 2015)

Furthermore, *SEM* analysis (Figure 4a) showed the damaged gluten structure, resulting in increased tan δ. tan δ is an indicator of protein quality (the higher the tan δ, the weaker the gluten network structure) (Kong et al., 2007).

### 1.5 Effect on thermal properties

Hydrolysis by proteases influenced wheat gluten thermal stability, determined by DSC. The glass transition temperature (Tg) is normally related to the protein thermal stability (more Tg values associated with enhanced wheat gluten thermal stability). Several studies were reported that alcalase significantly increases Tg values due to high quantities of exposed hydrophobic groups (Wang et al., 2017). Furthermore, Tg found to be lower, in excessive hydrolysis, due to splitting hydrophobic groups in to ionized groups. The change in enthalpy (ΔH) shows the extent of arrangements in the protein structure and straightly have relationship with its denaturation. Partially hydrolyzed gluten with alcalase significantly decreases ΔH parameter due to the alteration of the tertiary structure of gluten and less heat energy requirement (Wang, Qin, et al., 2017).

### 1.6 Effect on sensory characterization

Peptides with more hydrophobic amino acids are more likely to have lower bitter taste thresholds. When wheat gluten was hydrolyzed for 300 min by Proteax (a proteolytic enzyme obtained by *Aspergillus oryzae*), the hydrolyzed gluten protein showed minimum bitterness but maximum content of small peptides varying from 180 to 500 Da (He et al., 2019; Riu &Riu, 2016).

### 1.7 Effect on celiac disease

**FIGURE 3:**
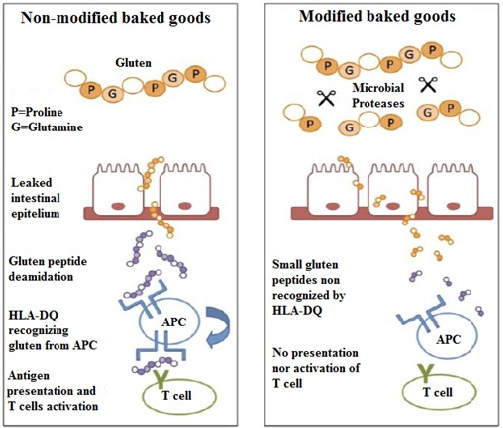
Adaptive immune response to nonmodified baked goods and nonactivation of T cells to modified baked goods by microbial proteases (Heredia‐Sandoval et al., 2016)

Uncontrolled immune response to wheat gluten causes a chronic enteropathy called celiac disease (CD) which refers to the pathology of the intestine. There are more than 60 immunogenic peptides in gluten derived from Triticum species. 33‐mer peptide with 13 proline residues and 10 glutamine residues is the most important immunogenic peptides, which is resistant to enzymatic proteolysis. Gliadin fractions and other wheat proteins can act as allergens; thus, celiac disease patients are not capable of tolerating these proteins (Heredia‐Sandoval et al., 2016; Bethune et al., 2006). Celiac disease patients carry HLA‐DQ2 and/or ‐DQ8 serotype which has the affinity to connect to antigens like gliadins and increase T‐cell‐mediated autoimmune reaction (Figure 3).

Studies have shown that various peptidases of fungal, plant, animal, or bacterial origin are able to hydrolyze gluten into harmless peptides. According to SDS‐PAGE pattern, proteolytic enzymes hydrolyze gliadins (Heredia‐Sandoval et al., 2016; Scherf et al., 2018; Socha et al., 2019; Wei et al., 2018, 2020). Bacterial peptidase (Krishnareddy & Green, 2017), fungal peptidase (Koning et al., 2005), and prolyl endopeptidases (PEPs) (Amador et al., 2019; Janssen et al., 2015; Kerpes et al., 2016; Mamo & Assefa, 2018) thoroughly degrade gliadin fractions to decrease gluten concentration and influence celiac disease. *Aspergillus niger* derived PEP (AN‐PEP) were assessed in clinical cases for their impact on modifying immune responses to gluten in celiac patients (Lähdeaho et al., 2014). Guerdrum and Bamforth (2012) reported that PEP addition in brewing technology decreased the prolamin and all of the identified immunopathogenic gluten epitopes in beer production (Akeroyd et al., 2016).

On the contrary, many of the recent investigations which employed enzyme‐linked immunosorbent assay (ELISA), mass spectrometry, and Western blot analysis reported that PEP did not thoroughly destroy the whole gluten proteins (Allred et al., 2017; Colgrave et al., 2017; Fiedler et al., 2018; Panda et al., 2015), which indicates that beers treated with PEP are not safe for CD patients.

### 1.8 Xylanase

Xylanases (EC 3.2.1.8) are able to break glycosidic linkages in arabinoxylans, producing smaller fragments. Xylanase from various sources has different mechanisms: 1) *Xylanase* obtained from *A. niger* degrades water‐extractable arabinoxylans exist in flour and reduces the molecular mass and dough viscosity of water‐extractable arabinoxylans; therefore, enhances the gluten agglomeration behavior and the larger gluten aggregates formation; 2) Xylanase generated from *B. subtilis* solubilizes water‐unextractable arabinoxylans augments the dough viscosity and negatively impacts gluten agglomeration (Romanowska et al., 2006). By removing arabinoxylans from gluten, xylanase alters the water distribution between gluten proteins and arabinoxylans, hence indirectly affect bread quality. Improving bread quality in the presence of xylanase also might be due to pentosan destruction and viscosity reduction effect of this enzyme. Viscosity reduction causes polymer chains to get next to each other easier, and the gluten aggregation would occur (Amiri et al., 2016). According to the competition between gluten and pentosan for water absorption, the degradation of pentosans by xylanase positively affects the gluten water binding characteristics (Figure 5; Amiri et al., 2016). On the contrary, some researchers found no evidence of xylanase removing arabinoxylans from gluten (Steffolani et al., 2010). These researchers believe that the evidence do not demonstrate the cleavage of covalent bonds between arabinoxylans and gluten by xylanase (Amiri, et al., 2016).

### 1.9 Effects on rheological properties

**FIGURE 4:**
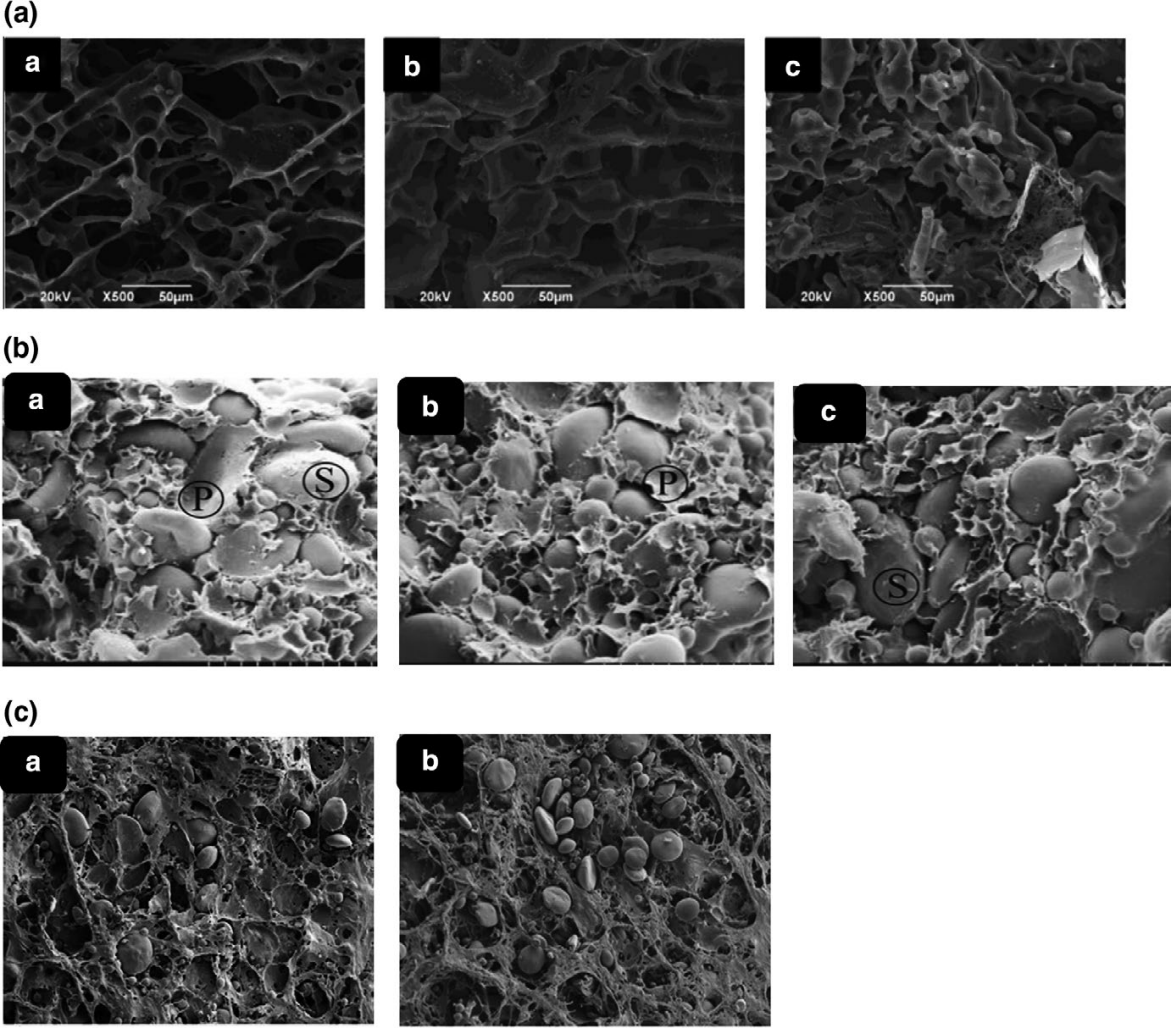
Scanning electron microscopy (*SEM*) of wheat gluten samples (A) WG (a), WG treated with desired quantity of Alcalase (0.04%, HWG‐4; b) and (0.10%, w/w, HWG‐10; c) (Wang et al., 2016). *SEM* of gluten (B) without enzymes (a), xylanase (b), and cellulase (c) (Wang et al., 2018). *SEM* images of dough treated with pentosanase (C), dough without pentosanase (a), and dough treated with pentosanase (b) (Steffolani et al., 2010) (Sun et al., 2019)

**FIGURE 5:**
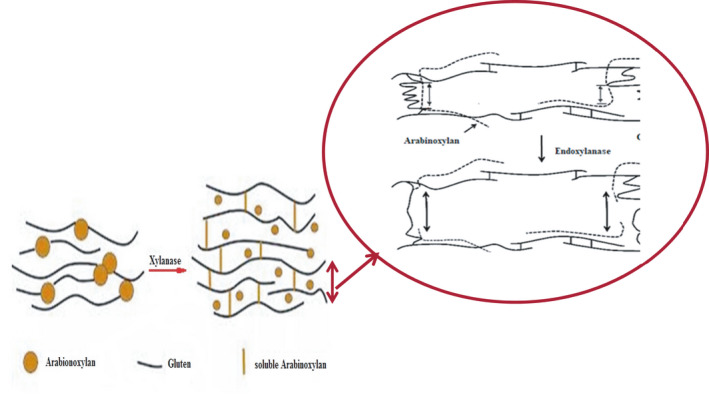
Xylanase reaction with gluten (Steffolani et al., 2010)

Steffolani et al., (2010) showed that endo‐xylanases increased SDS‐unextractable proteins, which can be explained by the importance of arabinoxylans in changing the extractability of the glutenin polymers (Figure 4b and Figure 5). Arabinoxylan breakdown with xylanase resulted in a less viscous dough, thereby augmenting the protein fragments mobility and facilitates their hydrophobic connections. These interactions would cause a more rapid protein aggregation which is due to the removing of steric interruption of arabinoxylans. Xylanases also redistributed water from arabinoxylans to the gluten and starch phase, make water more available to plasticize protein, thereby helping the gluten development. This made the dough and bread crumb softer and positively influenced bread making (Altınel & Ünal, 2017; Harada et al., 2000; Nevsky et al., 2018). In certain studies, the dough hardness was clearly reduced by adding high xylanase dosage to flour. The reduced dough hardening indicated the impact of xylanase on the interactions between glutenin and water soluble pentosans which leads to decrease disulfide cross‐linking and increase in SH content (Amiri, et al., 2016). Based on rheological results, xylanase tended to decrease storage modulus (G´) and augment tan δ which both inhibits less elastic behavior of dough in comparison with the control gluten samples. This phenomenon might be attributed to the degradation of pentosans and changing the gluten structure in samples treated with xylanase (Amiri et al., 2016; Steffolani et al., 2010). Moreover, the softening effect of xylanase is attributed to the release of water that occurs when arabinoxylans are hydrolyzed and the water binding capacity is reduced. The released water can reduce dough viscosity resulting in softer dough with better machining properties (Harada et al., 2000).

### 1.10 Pentosanase

Pentosanase is responsible for the conversion of water‐unextractable arabinoxylans to water‐extractable forms. Sun et al., (2019) revealed that water‐extractable arabinoxylans positively affect bread volume and textural properties, while water‐unextractable arabinoxylans cause undesirable product quality due to the competition for water and hinders gluten formation during the development of dough (Figures 2D and 4C). This conversion affects gluten network by five possible reactions: 1) Water‐unextractable arabinoxylans have high water‐holding capacity, which might cause lower water availability for gluten development, due to competition for water. Therefore, this conversion is known to improve gluten formation in baked products (Yang et al., 2017); 2) The pentosan–protein network will be weakened during the conversion of water‐unextractable arabinoxylans to their water‐extractable form. The cleavage of pentosan–protein bonds would release water, which is necessary for gluten development (Liu et al., 2018; Verjans et al., 2010); 3) Pentosanase would produce pentosans with smaller size which cause a redistribution of free water and reduce the steric hindrance of insoluble pentosans, thereby elevating the interaction between proteins (Steffolani et al., 2010); 4) Interaction among glutenin and water soluble pentosans lead to increase in SH content and dough softening according to SEM analysis (Figure 4C; Steffolani et al., 2010); 5) the enzyme enhanced gluten coagulation through reducing the steric impediment of the pentosans related to gluten and counteracting the gluten chemical aggregation (Steffolani et al., 2010).

### 1.11 Effects on rheological properties

Pentosanase produces a dough of greater extensibility and lower resistance to extension by interfering in protein–pentosan interactions (Primo‐Martin et al., 2003). Decrease in resistance values of doughs treated with pentosanase possibly is due to the increasing amount of soluble pentosans and released amount of free water and the inhibition of gluten network formation according to glutenin–pentosan interactions (Primo‐Martin et al., 2003). Revealed a decrease in development time and dough stability in pentosanase treated flours, which leads to production of weak doughs.

### 1.12 Cellulase

**FIGURE 6:**
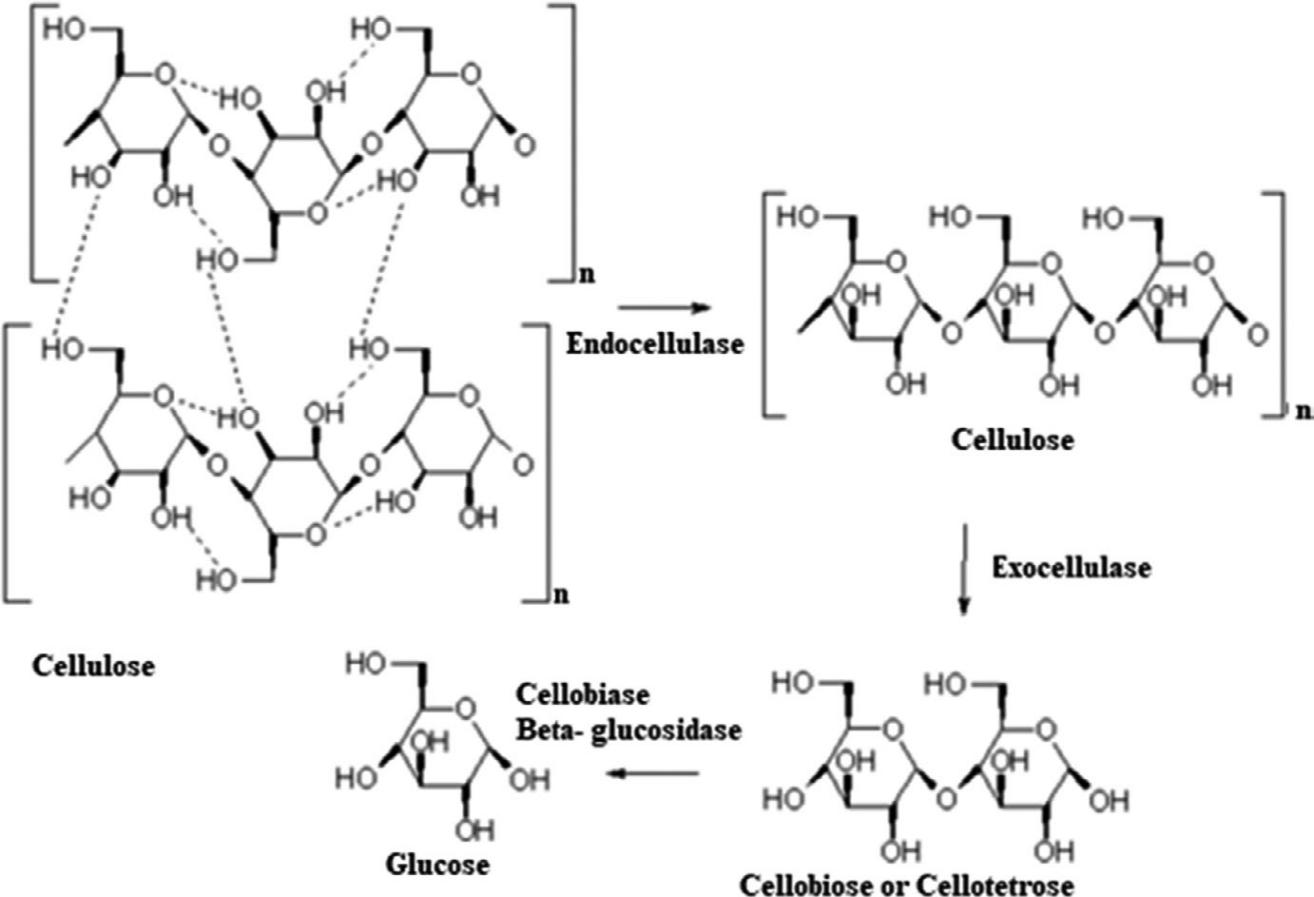
Cellulase mode of action

Cellulase (EC 3.2.1.4) is composed of enzymes which hydrolyze cellulose into cellobiose, glucose, and oligosaccharides (Figure 6) (Altınel & Ünal, 2017; Nigam, 2013; Park et al., 2019; Wang, Chen, et al., 2018; Vetrano et al., 2005). According to researches, cellulase used in bread dough resulted in a continuous gluten network (Wang, Chen, et al., 2018; Grigoras, 2017), and subsequently decrease in bread hardness and ameliorate the bread sensory evaluations (Yurdugul et al., 2012). The softening effects of cellulase on dough rheology also allow for a shorter baking time in certain baked goods like crackers (Carson, 2017).

Extensibility is an important factor reflecting the dough strength. Extensibility of wheat dough is closely related to the content of dietary fiber. According to Lu et al., (2015), cellulase could degrade dietary fiber and reduce the extensibility of wheat dough. Moreover, the softening mechanism of cellulase might be attributed to the release of water that occurs when cellulose, as cellulase substrate is hydrolyzed to reduce its water binding capacity. The released water can reduce dough viscosity resulting in softer dough with better machining properties (Figure 4b; Harada et al., 2000; Zhang et al., 2019). In contrast, according to Liu et al., (2017), the cellulase addition significantly (*p* <.05) increased the development time, stability, departure time, mixing tolerance index, extensibility, and stickiness of regular dough, and decreased both softening and resistance to extension.

The antistaling effect of cellulase on bread is described by Yurdugul et al., (2012). It can be noted that the cross‐linking between starch–protein is in charge of bread staling. The antistaling effect of cellulase could be according to the enzyme cell wall degradation, and monosaccharides and oligosaccharides resulting from the enzyme action which cause an alteration in water distribution between starch–protein matrix (Yurdugul et al., 2012; Decamps et al., 2016; Joye et al., 2009).

## 2 CONCLUSIONS

Adding enzymes to wheat flour has recently become a common practice to overcome the gluten deficiencies according to their impact on the properties of gluten protein and network construction through affecting its cross‐linking and bonds. Beside the alteration of gluten functionality, enzyme modification is recognized as a safer and healthier method compared with chemical agents because they are inactivated following the heating process in wheat‐based foods. Enzymatic hydrolysis strongly ameliorates the emulsification, solubility, foaming, and nutritive properties of gluten proteins. The final quality of bakery products significantly owes to flour composition (proteins, starch, arabinoxylan, pentosan, cellulose, etc) and their interactions. Having softening effects, hydrolytic enzymes affect flour ingredients and directly and indirectly affect gluten properties. Taken together, protease, peptidase, and alkalase directly affect gluten, while xylanase (affect arabinoxylan), pentosanase (affect pentosan), and cellulase (affect cellulose) indirectly affect gluten protein. It can be concluded that through enzyme modification, gluten characteristics can be favorably altered in wheat‐based products.

## 3 ETHICS STATEMENT

Human and animal testing is unnecessary in this study.

## CONFLICT OF INTEREST

The authors declare that they do not have any conflict of interest.

## AUTHOR CONTRIBUTION

**Kiana Pourmohammadi:** Conceptualization (equal); Formal analysis (equal); Investigation (equal); Project administration (equal); Visualization (equal); Writing‐original draft (equal); Writing‐review & editing (equal). **Elahe Abedi:** Conceptualization (equal); Investigation (equal); Methodology (equal); Project administration (equal); Writing‐review & editing (equal).
